# The Association of Upper Body Obesity with Insulin Resistance in the Newfoundland Population

**DOI:** 10.3390/ijerph18115858

**Published:** 2021-05-29

**Authors:** Sherif Youssef, Matthew Nelder, Guang Sun

**Affiliations:** Department of Medicine, Memorial University of Newfoundland, St. John’s, NL A1B 3V6, Canada; syoussef@mun.ca (S.Y.); mkn453@mun.ca (M.N.)

**Keywords:** obesity, metabolic syndrome, insulin resistance

## Abstract

Body-fat distribution is a primary risk factor for insulin resistance and cardiovascular disease. Visceral fat explains only a portion of this risk. The link between upper-body fat and insulin resistance is uncertain. Furthermore, upper-body fat is not clearly defined. Dual-energy X-ray absorptiometry (DXA) can accurately quantify body fat. In this study, we explored the relationship between non-visceral upper-body adiposity and insulin resistance and other markers of metabolic syndrome. Fat proportions in the upper body, leg, and visceral regions were quantified by using DXA in 2547 adult Newfoundlanders aged 19 and older. Adjusting for remaining fat regions, we performed partial correlation analysis for each body region and insulin resistance defined by the Homeostatic Model of Assessment (HOMA). Similarly, partial correlation analysis was also performed between each fat region and other markers of metabolic syndrome, including high-density lipoprotein cholesterol (HDL), triglycerides (TG), body mass index (BMI), and blood pressure. Major confounding factors, including age, caloric intake, and physical activity, were statistically controlled by using partial correlation analysis. Interactions between sex, menopausal status, and medication status were also tested. Arm adiposity was correlated with HOMA-IR (R = 0.132, *p* < 0.001) and HOMA-β (R = 0.134, *p* < 0.001). Visceral adiposity was correlated with HOMA-IR (R = 0.230, *p* < 0.001) and HOMA-β (R = 0.160, *p* < 0.001). No significant correlation between non-visceral trunk adiposity and insulin resistance was found. Non-visceral trunk adiposity was negatively correlated with HDL in men (R = −0.110, *p* < 0.001) and women (R = −0.117, *p* < 0.001). Non-visceral trunk adiposity was correlated with TG (total: R = 0.079, *p* < 0.001; men: R = 0.105, *p* = 0.012; women: R = 0.078, *p* = 0.001). In menopausal women, leg adiposity was negatively correlated with HOMA-IR (R = −0.196, *p* < 0.001) and HOMA-β (R = −0.101, *p* = 0.012). Upper-body adiposity in the arms is an independent contributor to insulin resistance. Upper-body adiposity in the non-visceral trunk region is an independent contributor to metabolic syndrome. Leg adiposity is protective against metabolic syndrome in women.

## 1. Introduction

From population-wide studies, it has become clearly evident that strong correlations between BMI and insulin resistance exist [1]. However, racial and ethnic differences with respect BMI limit its use in identifying individuals who are at risk of developing insulin resistance and metabolic syndrome [2]. Body-fat distribution has been thought to be associated with increased risk for developing insulin resistance, diabetes, metabolic syndrome, and cardiovascular disease [3,4,5,6]. One of the fat depots known to be associated with increased risk of insulin resistance and metabolic syndrome is visceral adiposity [7]. Although it is an important factor, visceral adiposity does not solely account for the development of insulin resistance [8]. Therefore, it has been hypothesized that perhaps body-fat depots other than visceral fat may also be linked with insulin resistance [9]. Based on smaller studies, non-visceral subcutaneous fat has been suggested to be linked with insulin resistance and metabolic syndrome [10], whereas other studies have suggested that lower extremity fat may be protective of insulin resistance and metabolic syndrome [11,12]. It is not yet known if upper-body fat accumulation in the arm and truncal regions (excluding visceral fat) is linked with insulin resistance. Furthermore, there is no clear definition for upper-body fat in the current literature.

Dual-energy X-ray absorptiometry (DXA) whole body scanning has become an established way of accurately quantifying body fat [13]. Compared with BMI, DXA allows accurate quantification of fat distribution and adiposity in the different body compartments [13]. Using this application of DXA would, thus, be theoretically advantageous in measuring the effects of subtle changes in body fat composition on insulin resistance and markers of metabolic syndrome such as fasting insulin, blood glucose, cholesterol, triglyceride levels, and blood pressure.

Insulin resistance can be measured by several methods. These methods include fasting insulin levels, glucose tolerance testing, and the hyperinsulinemic euglycemic clamp method. The gold standard method for measuring insulin resistance in both patients with or without diabetes is the hyperinsulinemic euglycemic clamp method. This method, however, is not only invasive and time-consuming but also requires methodologically rigorous protocols. Given the feasibility issues of the euglycemic clamp method, it is generally not practical for quantifying insulin resistance in the clinical setting or from a large number of individuals. Alternatively, the homeostasis method assessment of insulin resistance (HOMA-IR) has been developed as an alternative to the euglycemic clamp method (refer to methods sections for Equations). When measuring insulin resistance, researchers have found that fasting insulin levels and HOMA-IR correlate well with the gold standard hyperinsulinemic euglycemic clamp method [14].

Thus, using DXA as a means of quantifying upper-body fat and HOMA as a model for measuring insulin resistance, it may be possible to measure interactions between body-fat distribution based on DXA scanning and insulin resistance as measured by HOMA in a practical and feasible manner.

The primary purpose of this study was to explore associations between non-visceral upper-body adiposity, insulin resistance, and metabolic syndrome in the general adult population of Newfoundland. We hypothesized that similar to visceral adiposity, increased adiposity in the upper-body and lower-body compartments was associated with varying levels of insulin resistance and metabolic risk. Particularly, we hypothesized that increased upper-body adiposity, specifically in the arm and the non-visceral trunk regions would be associated with increased risk for insulin resistance and metabolic syndrome.

## 2. Materials and Methods

Ethics statement: This study was approved by the Health Research Ethics Authority (HREA) of Newfoundland, St. John’s, Canada, with project identification code 10.33. Written consent was provided by all who participated.

Study sample: 2547 Adults (1845 women and 702 men) from the Province of Newfoundland and Labrador (NL) were recruited for this study. The recruitment strategy included the use of flyers and advertisements which were displayed and readily available through brochures, bulletin boards, and newsletters throughout the Memorial University campus and the Health Sciences Centre in the city of St. John’s in Newfoundland. Recruited participants were also encouraged to invite their family members and friends through word-of-mouth to participate in this study. Recruitment took place from 2003 to 2017. Eligibility requirements were as follows:

1. Nineteen years of age or older.
2. Participants must have been born in NL, and the participant’s family must have been in NL for at least 3 generations.
3. Participants have no serious uncontrolled metabolic, endocrine, or cardiovascular conditions.
4. Participants must not be pregnant at the time of study.

Caloric intake and physical-activity assessment: Caloric intake was measured by using the Willett Food Frequency Questionnaire (FFQ) which measures an individual’s last 12 months of dietary behaviors [15,16]. The quantity of each food item was converted into a daily average intake value. This value was then entered in the Nutribase Clinical Nutrition Manager (software version 9.0; Cybersoft Inc., Phoenix, AZ, USA). We used the Baecke physical activity questionnaire to assess physical activity levels. This questionnaire measures physical activity by using three categories: work, sport, and leisure.

Body composition measurements and definitions: Body composition measurements which included trunk, android, visceral, gynoid, arm and leg fat were assessed using dual-energy X-ray absorptiometry (DXA; Lunar Prodigy; GE Medical Systems, Madison, WI, USA). Measurements were taken while patients lied horizontally on the DXA scanner following a 12 h fast. The trunk, arm, and leg regions were defined as per the DXA manufacturer’s specifications. The trunk region was defined as the area from the lowest boundary of the pelvis cut to the highest boundary of the neck [17,18,19,20]. Measurement of soft tissue mass and fat mass in each of these regions were obtained using the Lunar Prodigy software. The total body-fat percentage was calculated from the total body soft tissue mass. Similarly, this process was repeated to obtain the regional percentage of fat in the arm, leg, and trunk regions. Visceral fat mass and visceral fat volume on the other hand, were estimated using the DXA software algorithm. Estimating visceral fat mass by using DXA has been shown to be an accurate and valid method which correlates well with CT and MRI estimations of visceral fat mass and volume [21,22]. The estimated visceral fat mass was then subtracted from the total trunk soft tissue mass and also from the total trunk fat mass to obtain the total non-visceral trunk soft tissue mass and the total non-visceral trunk fat mass, respectively. The regional percentage of non-visceral trunk fat was calculated as the percentage of non-visceral trunk fat mass of the total non-visceral trunk soft tissue mass. Similarly, the regional percentage of visceral fat was calculated as the percentage of visceral fat mass of the total trunk soft tissue mass. Thus, the upper-body fat compartment comprised the regional arm-fat percentage and non-visceral trunk fat percentage, representing arm adiposity and non-visceral trunk adiposity, respectively. The visceral fat compartment comprised the regional visceral fat percentage, representing visceral adiposity. Finally, the lower-body fat compartment comprised the regional leg fat percentage, representing leg adiposity.

Metabolic marker measures: Serum concentrations of glucose, HDL cholesterol, and triglycerides were measured by using the Lx20 analyzer (Beckman Coulter Inc., Fullerton, CA, USA) with Synchron reagents. Serum insulin was measured by using the Immulite Immunoassay analyzer. Casual systolic and diastolic blood pressure measurements were obtained from participants. Insulin resistance and beta cell function were calculated by using the following HOMA Equations (1) and (2) [14]:

HOMA-IR = Fasting Plasma Insulin (mIU/L) × Fasting Plasma Glucose (mmol/L)/22.5(1)

HOMA-β = 20 × Fasting Plasma Insulin (mIU/L)/Fasting Plasma Glucose (mmol/L) − 3.5(2)

Statistical analysis: All statistical analysis was completed by using SPSS ver.23. The level of statistical significance was set at *p*-value < 0.05. Pearson partial correlation analysis was used to identify statistically significant correlations between the metabolic markers (Insulin, HOMA-β, HOMA-IR, TG, HDL, and casual systolic and diastolic blood pressure) and the potential confounding variables (age, physical activity, caloric intake, % carbohydrate intake, % fat intake, % protein intake, and % alcohol intake). Age, physical activity, and caloric intake were identified to have statistically significant correlations with the metabolic markers and, thus, were identified as confounding variables.

Pearson partial correlation analysis was then used to identify the correlations between each of the defined fat regions (non-visceral trunk adiposity, visceral adiposity, arm adiposity, and leg adiposity) and each of the metabolic markers (Insulin, HOMA-β, HOMA-IR, TG, HDL, and casual systolic and diastolic blood pressure). The identified confounding variables which included age, physical activity, and caloric intake were statistically controlled for in the correlation analysis. In addition, to adjust for potential confounding from the defined fat regions, we statically controlled the remaining defined fat regions when we analyzed each defined fat region.

Therefore, for example, the Pearson partial correlation analysis between arm adiposity and insulin resistance was determined controlling for non-visceral trunk adiposity, visceral adiposity, leg adiposity, age, physical activity, and caloric intake. This method of analysis was similarly applied to each of the defined fat regions and the remaining metabolic markers.

All results were then stratified by sex (male or female), menopausal status (premenopausal or menopausal), and medication status (taking medications or not taking any medications).

## 3. Results

Characteristics of participants: The study sample included a reasonably healthy adult Newfoundlanders from varying age groups. The vast majority of participants was European Caucasian of English and Irish descent. Most participants had good socioeconomic status, income security, and high level of education. The average age of women and men in our study was 43.9 years and 40.1 years, respectively. Men were significantly taller, heavier and by extension had higher BMIs than the women in our study. Women, on average, had much higher body-fat percentage than men. Women had an average of 37.3% body fat, while men had 25.3% body fat. Men had significantly higher waist circumferences than women; however, the hip circumferences were similar. All physical characteristic measurements are displayed in Table 1, Table 2 and Table 3.

Regional adiposity and insulin resistance: Arm adiposity, in the total population, was significantly correlated with both HOMA-IR (r = 0.132, *p* < 0.001) and HOMA-β (r = 0.134, *p* < 0.001) (Table 4). Visceral adiposity in the total population was also significantly correlated with both HOMA-IR (r = 0.230, *p* < 0.001) and HOMA-β (r = 0.160, *p* < 0.001) (Table 4). Non-visceral trunk adiposity was not significantly correlated with either HOMA-IR or HOMA-β (Table 4). Leg adiposity was significantly negatively correlated with HOMA-IR (r = −0.107, *p* < 0.001), and with HOMA-β (r = −0.044, *p* = 0.038) (Table 4).

In men, both arm and visceral adiposity were significantly correlated with both HOMA-IR and HOMA-β. The correlation between arm adiposity and HOMA-IR was 0.129 (*p* = 0.002), and HOMA-β was 0.186 (*p* < 0.001) (Table 5). The correlation between visceral adiposity and HOMA-IR was 0.240 (*p* < 0.001), and HOMA-β was 0.132 (*p* = 0.002) (Table 5). No significant correlations were observed between leg adiposity and non-visceral trunk adiposity and HOMA-IR or HOMA-β in men (Table 5).

Similarly, in women, both arm and visceral adiposity were significantly correlated with both HOMA-IR and HOMA-β. The correlation between arm adiposity and HOMA-IR was 0.114 (*p* < 0.001), and HOMA-β was 0.113 (*p* < 0.001) (Table 6). The correlation between visceral adiposity and HOMA-IR was 0.188 (*p* < 0.001), and HOMA-β was 0.126 (*p* < 0.001) (Table 6). In women, leg adiposity was significantly negatively correlated with HOMA-IR but not HOMA-β. The correlation between leg adiposity and HOMA-IR was −0.135 (*p* < 0.001) (Table 6). Similar to men, no significant correlation was observed between non-visceral trunk adiposity and HOMA-IR or HOMA-β in women (Table 6).

In premenopausal women, arm and visceral adiposity were both significantly correlated with both HOMA-IR and HOMA-β; however, non-visceral trunk adiposity and leg adiposity were not (Table 7). In menopausal women, a similar trend was observed, however, leg adiposity was significantly negatively correlated with both HOMA-IR and HOMA-β (Table 8). In menopausal women, the correlation between leg adiposity and HOMA-IR was −0.196 (*p* < 0.001), and HOMA-β was −0.101 (*p* = 0.012) (Table 8).

Separating the results by those using medications and those not using medications, similar correlations between arm, visceral adiposity, and HOMA-IR and HOMA-β were observed as those in the total population (Table 9 and Table 10). Leg adiposity was significantly negatively correlated with both HOMA-IR and HOMA-β in participants using medications but not in participants not using medications. In participants using medications, the correlation between leg adiposity and HOMA-IR was −0.135 (*p* < 0.001), and HOMA-β was −0.084 (*p* = 0.03) (Table 9).

Regional adiposity and HDL: Non-visceral trunk adiposity in the total population was significantly negatively correlated with HDL (r = −0.149, *p* < 0.001) (Table 4). This correlation was also observed for men (r = −0.110, *p* = 0.008) and women (r = −0.117, *p* < 0.001) when results were separated by sex (Table 5 and Table 6). Visceral adiposity in the total population was also significantly negatively correlated with HDL (r = −0.316, *p* < 0.001) (Table 4). Similarly, this correlation was also observed for men (r = −0.167, *p* < 0.001) and women (r = −0.250, *p* < 0.001) when results were separated by sex (Table 5 and Table 6). Although arm adiposity in the total population was significantly positively correlated with HDL, the size of this correlation was small (r = 0.080, *p* < 0.001), and furthermore, this relationship was not statistically significant when men and women were separated (Table 4, Table 5 and Table 6). Leg adiposity was significantly correlated with HDL in the total population (r = 0.128, *p* < 0.001) and in women (r = 0.083, *p* < 0.001); however, this correlation was not observed in men (Table 4, Table 5 and Table 6). Separating results by menopausal status, leg adiposity was significantly correlated with HDL in menopausal women (r = 0.126, *p* < 0.001), but not premenopausal women. (Table 7 and Table 8). When results were separated by medication use, similar relationships were observed between the different adiposity regions and HDL (Table 9 and Table 10).

Regional adiposity and TG: Non-visceral trunk adiposity was significantly correlated with TG in the total population (r = 0.079, *p* < 0.001) (Table 4). This relationship was similarly observed across most groups (Table 5, Table 6, Table 7, Table 8, Table 9 and Table 10). Visceral adiposity was significantly correlated with TG in the total population (r = 0.313, *p* < 0.001) (Table 4). This relationship was maintained regardless of sex, menopausal status, and medication use (Table 5, Table 6, Table 7, Table 8, Table 9 and Table 10). Leg adiposity was significantly negatively correlated with TG in the total population (r = −0.093, *p* < 0.001) (Table 4). This relationship was similarly observed across most groups (Table 5, Table 6, Table 7, Table 8, Table 9 and Table 10). Arm adiposity was not significantly correlated with TG in total population or any group (Table 4, Table 5, Table 6, Table 7, Table 8, Table 9 and Table 10).

Regional adiposity and blood pressure: Visceral and non-visceral trunk adiposity were significantly correlated with casual systolic and diastolic blood pressure in the total population (Table 4). Arm adiposity was not significantly correlated with casual systolic and diastolic blood pressure in the total population (Table 4). Leg adiposity was significantly negatively correlated with casual systolic and diastolic blood pressures in the total population (Table 4).

Regional adiposity and BMI: All adiposity regions were significantly correlated with BMI in the total population and across most groups (Table 4, Table 5, Table 6, Table 7, Table 8, Table 9 and Table 10). Non-visceral trunk adiposity was not significantly correlated with BMI in premenopausal women (Table 7). Leg adiposity was not significantly correlated with BMI in men (Table 5).

## 4. Discussion

According to the metabolic syndrome definition, central body adiposity has been linked to increased insulin resistance and increased cardiometabolic risk [23]. Central body adiposity includes body fat in the visceral and non-visceral trunk regions. One of the body-fat depots thought to pose these increased risks is visceral fat [24]. However, on its own, visceral fat accounts for only a proportion of this increased risk. Furthermore, the link between adiposity in different body regions and insulin resistance has remained poorly understood. Although obesity has been linked to insulin resistance and metabolic syndrome, many individuals with BMI measurements which do not meet the clinical definition of obesity are at increased cardiometabolic risk [25]. In this study we aimed to further elucidate some of the links between regional body adiposity and insulin resistance and markers of metabolic syndrome, irrespective of BMI. Establishing these links may help in identifying non-obese individuals who are at increased cardiometabolic risk.

In our cross-sectional study, we defined body adiposity in the three main body compartments, using DXA. The upper-body fat compartment comprised the proportion of fat in the arm and non-visceral trunk region, representing arm adiposity and non-visceral trunk adiposity, respectively. The visceral fat compartment comprised the proportion of fat in the visceral trunk region, representing visceral adiposity. Finally, the lower-body fat compartment comprised the proportion of fat in the leg region, representing leg adiposity. Our definition of regional adiposity was based on the proportion of fat mass in the defined region relative to the total soft tissue mass within that respective region. Given that our study included over 2500 Newfoundland adults with varying body characteristics, this definition allowed for the standardization of regional adiposity regardless of total body-fat mass, total body weight, differences in anthropometric measurements (hip and waist circumference), and BMI.

In our study, we aimed to identify associations between the different adiposity regions and insulin resistance as defined by HOMA, and the metabolic markers including HDL, TG, and casual systolic and diastolic blood pressure.

Our primary finding from this study showed that increased arm adiposity is significantly correlated with higher HOMA values and, therefore, increased insulin resistance in both men and women. Contrary to this, Vasan et al. have found that, in men, increased arm fat adjusted for BMI and fat-mass index (FMI) decreased the odds of insulin resistance and CVD risk factors [26]. While no exact mechanism between arm adiposity and insulin resistance or CVD risk factors has been described, this study goes on to propose that this may be because the arm region in men acts as a “reservoir” of subcutaneous fat storage, similar to the function of leg adiposity in women. The study by Vasan et al. includes men ages 29–55, while ours includes individuals of a much broader age range which may account for some differences in our findings. Data from 9971 individuals aged 18 and over, from the National Health and Nutrition Examination Survey, found that middle upper-arm circumference, an index of arm adiposity, was significantly associated with metabolic syndrome as defined by using the National Cholesterol Education Program Adult Treatment Panel III criteria and could possibly serve as a reliable screening tool for metabolic syndrome [27]. Our primary finding remained statistically significant after rigorously controlling for several confounding factors including the remaining adiposity regions and visceral adiposity. Thus, these results support our hypothesis that increased adiposity in the upper-body fat compartment, specifically in the arm region, is associated with increased insulin resistance.

**Figure 1:**
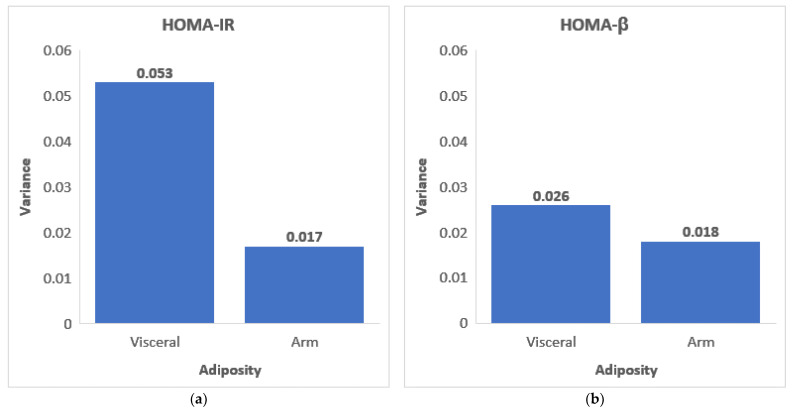
Visceral and arm adiposity variance R^2^ in (**a**) HOMA-IR and (**b**) HOMA-β.

In a similar manner of analysis, our findings also showed that visceral adiposity was also associated with increased insulin resistance, which is congruent with previous research [28,29]. In our model, visceral adiposity accounted for 5.3% of the variance in HOMA-IR (r^2^ = 0.053) and 2.6% of the variance in HOMA-β (r^2^ = 0.026) (Figure 1). Comparatively, arm adiposity in the upper-body compartment accounted for 1.7% of the variance in HOMA-IR (r^2^ = 0.017) and 1.8% of the variance in HOMA- β (r^2^ = 0.018) (Figure 1). Thus, although arm adiposity and visceral adiposity may be contributing to insulin resistance, the main contributors to insulin resistance remain unknown.

We did not observe any associations between non-visceral trunk adiposity in the upper-body compartment and insulin resistance. However, similar to other studies, we did find an inverse relationship between leg adiposity and insulin resistance suggesting that lower-body adiposity may have a protective effect [30,31]. Among women, an inverse relationship between leg adiposity and HOMA-IR was observed, but the correlation between leg adiposity and HOMA-β remained insignificant. It should be noted that HOMA-β is a measure of insulin secretion and its relationship to glucose metabolism can sometimes be difficult to interpret, particularly in cross-sectional studies such as ours where directionality cannot be concluded. For example, in an individual with prediabetes, HOMA-β may be higher, indicating that insulin secretion is increasing to compensate for higher blood sugar [32]. However, among patients with long-standing diabetes, HOMA-β may be lower because beta-cell damage has happened [32]. In this case, adiposity in the lower-body compartment seems to have a favorable effect on insulin sensitivity perhaps suggesting that the compensatory mechanism of insulin is not as pronounced among women with more adiposity in the lower-body compartment.

Unlike leg adiposity, we did not observe sex-specific differences with regards to increased arm adiposity and insulin resistance. This finding is consistent with another cross-sectional study which found that large mid-upper-arm circumference is associated with metabolic syndrome in adults after adjusting for gender and age [33].

In addition to markers of insulin resistance, we also examined several lipid profile markers associated with cardiometabolic risk. HDL is an important measure of cardiometabolic risk, as it is known to be protective against CVD [34]. Lower levels of HDL may result in a higher risk of developing metabolic syndrome among other metabolic issues [35,36]. HDL levels are also one of the main criteria used in the diagnosis of metabolic syndrome [37]. Our investigation revealed that increased non-visceral trunk adiposity exhibited a significant inverse correlation with HDL. This correlation was observed in the total population and among menopausal women. Before women reach menopause, subcutaneous fat stores tend to build in the lower-body area rather than the abdominal area; however, this balance seems to shift towards abdominal fat storage after menopause. One study which took care to screen for hormone replacement therapy, birth control usage, and drugs that could affect lipid metabolism found that, compared with peri-menopausal women, menopausal women (matched for BMI, body fat, and race) exhibited significantly lower lipoprotein lipase activity in the abdominal area leading to increased accumulation of abdominal fat [38]. A study by Pedersen et al. provided evidence that the usual female body-fat distribution is maintained in part by female sex hormones, specifically estradiol, which inhibits lipolysis in certain subcutaneous fat regions by directly increasing the number of antilipolytic α2A-adrenergic receptors in subcutaneous adipocytes [39]. It is possible that this antilipolytic activity is more pronounced in the lower body compartment in premenopausal women, favoring a body-fat distribution pattern that contributes to healthier lipid profiles [39].

Research has shown that increased TG can significantly increase risk of heart attack and stroke [40,41]. In our study, non-visceral upper-body adiposity also showed a significant association with TG. This relationship was detected in the total population and in both men and women. Non-visceral trunk adiposity in men exhibited a higher correlation with TG compared to that seen in women (r = 0.105 vs. r = 0.078). One potential reason for this observation may be that, in some women, a predisposition to lower-body fat accumulation, may mitigate some of the detrimental effects of subcutaneous upper-body adiposity. Lower-body adiposity is thought to provide systemically beneficial effects on lipid profiles and other cardiometabolic risk factors [42]. This benefit is thought to be due to the unique activity of lipoprotein lipase in the gynoid adipocytes that results in more storage and less release of free chain fatty acids into the systemic circulation, leading to improved downstream metabolic health effects [43].

We also observed that visceral and non-visceral trunk adiposity were associated with increased casual systolic and diastolic blood pressure which was consistent with other studies. A study of 2082 men and 3146 women over 18 years of age, with no history of diabetes, hypertension, ischemic heart disease, or chronic obstructive pulmonary disease, also found that truncal obesity was significantly associated with higher systolic blood pressure [44]. A study by Zhang et al. concluded that leg fat was inversely associated with high blood pressure among a diverse group of 8802 individuals who participated in the 1999–2004 US National Health and Nutrition Examination Survey [45]. Both these studies took part in large, diverse, US populations consisting of individuals of several ethnicities. We do take care to note that our findings, as with other studies, were based on casual blood pressure readings as opposed to the formal diagnosis of hypertension as defined by the metabolic syndrome.

Therefore, our findings suggest that, although non-visceral trunk adiposity was not significantly correlated with insulin resistance, it may still be an important contributor to HDL and TG which are markers of cardiovascular risk and metabolic syndrome.

While the findings of our study have supported our hypothesis that non-visceral upper-body adiposity may be associated with insulin resistance and markers of metabolic syndrome, there are limitations that must be acknowledged prior to drawing reliable conclusions from our findings. Firstly, our cross-sectional study was mainly intended to explore associations and not ascertain causality between body adiposity and metabolic markers. Secondly, participant recruitment was completed through convenience sampling throughout the Memorial University of Newfoundland campus and Health Sciences Centre in St. John’s Newfoundland. This method likely generated a sample of relatively healthy adults with good socioeconomic status and high education levels. As a result, the applicability of our findings to the broader demographic in Newfoundland may be limited. Thirdly, we acknowledge that our sample had more female than male participants. However, this skew was not intentional as our recruitment process did not target or favor a specific gender. Despite this limitation, our sample was sufficiently large, and we took care to analyze our results according to each group separately.

Fourthly, there were a few cases where data regarding insulin measurements were not detected by the insulin measurement kits. As a result, these data points were recorded as missing. However, these cases were few compared to the remainder of our large dataset and did not change our reported findings. Fifthly, two of the control variables used in this study, specifically physical activity and caloric intake, were based on questionnaires given to each participant. While each questionnaire was designed to best capture long-term eating and physical activity behaviors, it is subject to possible recall bias and self-reporting bias, as are many questionnaires. Additionally, it is well-known that many common medications, including antihypertensive agents, lipid-lowering agents, antiglycemic agents, and hormonal treatments, have known and important impacts on metabolic risk factors. In our study, data collection regarding specific medications was not possible due to inaccuracies in recall or incomplete documentation of medications. As a result, making definitive conclusions about the effect of participant medication use was limited to broad and general observations. In general, we observed that participants using medications tended to be slightly older and had a higher BMI (refer to Table 3).

Other possible limitations in our study are the technical limits of the DXA machinery and accompanying software. For example, the truncal region of the body as defined by our DXA scanner includes a very small portion of the hip and thigh; however, we did correct this to the best of our ability. It is also important to note that we are reporting correlations, and while we have taken great care to control for possible confounders, further research is required before we can conclude that any of the relationships observed are causal. Moreover, given that the determinants of insulin resistance and metabolic syndrome are still not entirely known, the different adiposity regions should not be solely relied on to predict insulin resistance and metabolic syndrome in the clinical setting.

Despite these limitations in our study, there were several strengths which greatly lend to the reliability and accuracy of our findings. Firstly, we used DXA as a way to accurately quantify body composition. Given its safety, reliability, accuracy, and cost-effectiveness, DXA scanning is considered the gold standard for body composition measurements [19]. DXA scanning shares a significant concordance with much more invasive, expensive scanning techniques such as CT or MRI scanning [19]. Despite these considerations, it is important to note that DXA may not be readily available or widely used for quantifying body fat or predicting metabolic risk in the non-research or clinical setting. Anthropometric indices including mid-upper-arm circumference and triceps skinfold thickness have been shown in some studies to correlate with metabolic risk and can perhaps be used as surrogate measures for arm adiposity in the clinical setting [27,46]. However, anthropometric surrogate measures for non-visceral trunk adiposity are not described in the literature.

Secondly, our sample in this study consisted of a large number of individuals from a general population who are all, at least, third generation Newfoundlanders. These screening measures helped ensure that our large sample was representative of the adult population of Newfoundland. Previous studies on the genetic substructure and the extent of the homogeneity among Newfoundlanders have found that this particular population is indeed relatively homogenous [47,48]. From our sample, the vast majority of participants was European Caucasian from English/Irish descent, which is consistent with findings from prior genetic studies [47,48]. Moreover, these studies have shown that the Newfoundland population is also relatively homogeneous with respect to dietary culture [47,48]. Although this homogeneity helped reduce some of the potential confounding effects in our analysis, it also reduced the generalizability of our findings to other populations.

We have also taken special care to ensure that our participants did not suffer from any serious metabolic disease at the time of participation to limit the possibility of extreme outliers which could potentially skew results. Additionally, we have noted the menopausal status of female participants to control for the metabolic differences often observed between pre-menopausal and menopausal women [49].

## 5. Conclusions

In conclusion, upper-body adiposity in the arm region may be an independent contributor to insulin resistance. Upper-body adiposity in the non-visceral trunk region is associated with reduced HDL and increased TG, respectively. Lower-body adiposity may be protective against metabolic syndrome in women. Our results aid in clarifying some of the effects of non-visceral upper-body adiposity with respect to metabolic risk. Establishing a link between upper-body adiposity and metabolic risk factors, independent of BMI, may be helpful in identifying at-risk individuals and preventing cardiometabolic diseases. However, additional studies are required to confirm our findings to establish a more causal relationship between regional adiposity, and insulin resistance and markers of metabolic syndrome. Although DXA may not be readily available outside of the research setting, surrogate anthropometric measures which correlate with upper-body adiposity can be further studied in the clinical setting.

## Figures and Tables

**Table 1:** Physical and metabolic characteristics of men and women.

Total	Women		Men	
N = 2547	Mean ± SD		Mean ± SD	
	N = 1845		N = 702	
Age *	43.87	±12.74	40.11	±14.04
Height (cm) *	162.50	±6.01	176.80	±6.68
Weight (kg) *	69.62	±14.36	87.31	±16.42
BMI *	26.35	±5.31	27.67	±4.62
Total Body Fat (%) *	37.27	±7.92	25.34	±8.24
Waist (cm) *	90.02	±14.38	98.15	±13.85
Hip (cm) *	100.66	±12.50	100.34	±10.81
Systolic BP (mm Hg) *	121.12	±16.19	131.52	±15.15
Diastolic BP (mm Hg) *	79.88	±11.03	83.16	±10.92
Physical Activity *	8.17	±1.56	8.53	±1.64
Calories *	1886.52	±928.73	2308.06	±1122.53
Insulin *	69.80	±68.164	78.11	±63.37
Glucose *	5.05	±0.80	5.34	±1.15
HOMA-IR *	2.39	±3.20	2.83	±3.48
HOMA-β *	136.64	±110.03	129.54	±105.05
HDL *	1.54	±0.38	1.21	±0.28
TG *	1.13	±0.68	1.38	±0.97

* *p* < 0.05; BMI, body mass index; BP, blood pressure; HOMA-IR, Homeostatic Model of Assessment Insulin Resistance; HOMA-β, Homeostatic Model of Assessment β-cell function; HDL, high-density lipoprotein; TG, triglycerides.

**Table 2:** Physical and metabolic characteristics of premenopausal and menopausal women.

Women	Premenopausal		Menopausal	
N = 1845	Mean ± SD		Mean ± SD	
	N = 1017		N = 828	
Age *	37.55	±10.10	53.86	±9.25
Height (cm) *	163.20	±5.81	161.19	±5.89
Weight (kg) *	68.91	±14.05	70.49	±14.52
BMI *	25.88	±5.19	27.06	±5.41
Total Body Fat (%) *	36.09	±8.09	39.19	±7.12
Waist (cm) *	87.75	±13.91	93.49	±14.48
Hip (cm) *	99.10	±12.61	103.24	±11.87
Systolic BP (mm Hg) *	119.07	±14.86	124.47	±17.27
Diastolic BP (mm Hg) *	79.17	±11.42	81.21	±10.38
Physical Activity *	8.37	±1.53	7.85	±1.53
Calories *	1919.20	±868.82	1806.19	±914.13
Insulin *	64.70	±44.36	76.49	±94.63
Glucose *	4.91	±0.63	5.25	±1.01
HOMA-IR *	2.10	±1.65	2.77	±4.74
HOMA-β *	141.12	±113.49	129.55	±109.82
HDL *	1.55	±0.38	1.54	±0.39
TG *	1.04	±0.64	1.28	±0.73

* *p* < 0.05; BMI, body mass index; BP, blood pressure; HOMA-IR, Homeostatic Model of Assessment Insulin Resistance; HOMA-β, Homeostatic Model of Assessment β-cell function; HDL, high-density lipoprotein; TG, triglycerides.

**Table 3:** Physical and metabolic characteristics of medicated and non-medicated participants.

Medication Status	Medications		No Medications	
N = 2547	Mean ± SD		Mean ± SD	
	N = 1407		N = 1140	
Age *	44.95	±13.68	40.62	±12.32
Height (cm) *	164.93	±7.89	168.08	±9.49
Weight (kg) *	74.39	±17.10	74.39	±16.29
BMI *	27.20	±5.55	26.18	±4.62
Total Body Fat (%) *	35.84	±9.00	31.71	±9.91
Waist (cm) *	93.35	±15.53	90.82	±13.26
Hip (cm) *	101.88	±12.73	98.80	±10.91
Systolic BP (mm Hg) *	124.71	±17.06	123.20	±16.04
Diastolic BP (mm Hg) *	81.47	±11.48	80.07	±10.69
Physical Activity *	8.10	±1.58	8.47	±1.59
Calories *	1954.33	±977.15	2062.31	±1039.05
Insulin *	78.37	±79.55	64.48	±45.68
Glucose *	5.21	±1.10	5.03	±0.58
HOMA-IR *	2.81	±4.12	2.15	±1.85
HOMA-β *	141.64	±108.70	126.64	±109.39
HDL *	1.46	±0.39	1.43	±0.38
TG *	1.29	±0.80	1.10	±0.76

* *p* < 0.05; BMI, body mass index; BP, blood pressure; HOMA-IR, Homeostatic Model of Assessment Insulin Resistance; HOMA-β, Homeostatic Model of Assessment β-Cell function; HDL, high-density lipoprotein; TG, triglycerides.

**Table 4:** Associations between regional adiposity and metabolic characteristics in the total population.

N = 2547	Visceral		Arm		Trunk		Leg	
	r	*p*-Value	R	*p*-Value	r	*p*-Value	R	*p*-Value
**HOMA-IR**	0.230	<0.001 *	0.132	<0.001 *	0.003	0.879	−0.107	<0.001 *
**HOMA-β**	0.160	<0.001 *	0.134	<0.001 *	−0.011	0.616	−0.044	0.038 *
**BMI**	0.521	<0.001 *	0.065	<0.001 *	0.203	<0.001 *	0.040	0.059
**HDL**	−0.316	<0.001 *	0.080	<0.001 *	−0.149	<0.001 *	0.128	<0.001 *
**TG**	0.313	<0.001 *	0.009	0.676	0.079	<0.001 *	−0.093	<0.001 *
**Systolic BP**	0.188	<0.001 *	0.014	0.498	0.088	<0.001 *	−0.135	<0.001 *
**Diastolic BP**	0.166	<0.001 *	0.021	0.320	0.100	<0.001 *	−0.102	<0.001 *

* *p* < 0.05; BMI, body mass index; BP, blood pressure; HOMA-IR, Homeostatic Model of Assessment Insulin Resistance; HOMA-β, Homeostatic Model of Assessment β-cell function; HDL, high-density lipoprotein; TG, triglycerides.

**Table 5:** Associations between regional adiposity and metabolic characteristics in men.

N = 702	Visceral		Arm		Trunk		Leg	
	r	*p*-Value	R	*p*-Value	r	*p*-Value	r	*p*-Value
**HOMA-IR**	0.240	<0.001 *	0.129	0.002 *	−0.069	0.097	−0.023	0.576
**HOMA-β**	0.132	0.002 *	0.186	<0.001 *	−0.027	0.518	−0.047	0.267
**BMI**	0.378	<0.001 *	0.244	<0.001 *	0.157	<0.001 *	0.013	0.756
**HDL**	−0.167	<0.001 *	0.009	0.839	−0.110	0.008 *	0.046	0.274
**TG**	0.219	<0.001 *	0.028	0.500	0.105	0.012 *	−0.104	0.013 *
**Systolic BP**	0.038	0.362	0.179	<0.001 *	−0.036	0.393	−0.082	0.05 *
**Diastolic BP**	0.083	0.047 *	0.135	<0.001 *	0.063	0.131	−0.120	0.004 *

* *p* < 0.05; BMI, body mass index; BP, blood pressure; HOMA-IR, Homeostatic Model of Assessment Insulin Resistance; HOMA-β, Homeostatic Model of Assessment β-cell function; HDL, high-density lipoprotein; TG, triglycerides.

**Table 6:** Associations between regional adiposity and metabolic characteristics in women.

N = 1845	Visceral		Arm		Trunk		Leg	
	r	*p*-Value	R	*p*-Value	r	*p*-Value	R	*p*-Value
**HOMA-IR**	0.188	<0.001 *	0.114	<0.001 *	0.026	0.286	−0.135	<0.001 *
**HOMA-β**	0.126	<0.001 *	0.113	<0.001 *	−0.009	0.712	−0.047	0.058
**BMI**	0.369	<0.001 *	0.279	<0.001 *	0.126	<0.001 *	0.224	<0.001 *
**HDL**	−0.250	<0.001 *	0.001	0.971	−0.117	<0.001 *	0.083	<0.001 *
**TG**	0.316	<0.001 *	−0.008	0.736	0.078	<0.001 *	−0.104	<0.001 *
**Systolic BP**	0.115	<0.001 *	0.074	0.003 *	0.075	0.002 *	−0.081	<0.001 *
**Diastolic BP**	0.103	<0.001 *	0.052	0.035 *	0.082	<0.001 *	−0.058	0.018 *

* *p* < 0.05; BMI, body mass index; BP, blood pressure; HOMA-IR, Homeostatic Model of Assessment Insulin Resistance; HOMA-β, Homeostatic Model of Assessment β-cell function; HDL, high-density lipoprotein; TG, triglycerides.

**Table 7:** Associations between regional adiposity and metabolic characteristics in premenopausal women.

N = 1017	Visceral		Arm		Trunk		Leg	
	r	*p*-Value	R	*p*-Value	r	*p*-Value	r	*p*-Value
**HOMA-IR**	0.341	<0.001 *	0.113	<0.001 *	−0.020	0.547	−0.044	0.179
**HOMA-β**	0.155	<0.001 *	0.082	0.012 *	−0.024	0.464	−0.004	0.910
**BMI**	0.462	<0.001 *	0.268	<0.001 *	0.038	0.247	0.314	<0.001 *
**HDL**	−0.274	<0.001 *	0.030	0.358	−0.073	0.026 *	0.043	0.188
**TG**	0.300	<0.001 *	0.001	0.985	0.031	0.352	−0.054	0.101
**Systolic BP**	0.148	<0.001 *	0.103	0.002 *	0.027	0.418	−0.059	0.073
**Diastolic BP**	0.118	<0.001 *	0.069	0.036 *	0.053	0.109	−0.040	0.227

* *p* < 0.05; BMI, body mass index; BP, blood pressure; HOMA-IR, Homeostatic Model of Assessment Insulin Resistance; HOMA-β, Homeostatic Model of Assessment β-cell function; HDL, high-density lipoprotein; TG, triglycerides.

**Table 8:** Associations between regional adiposity and metabolic characteristics in menopausal women.

N = 828	Visceral		Arm		Trunk		Leg	
	r	*p*-Value	R	*p*-Value	r	*p*-Value	R	*p*-Value
**HOMA-IR**	0.138	<0.001 *	0.128	<0.001 *	0.032	0.425	−0.196	<0.001 *
**HOMA-β**	0.112	0.005 *	0.122	0.002 *	−0.017	0.676	−0.101	0.012 *
**BMI**	0.283	<0.001 *	0.252	<0.001 *	0.207	<0.001 *	0.112	0.005 *
**HDL**	−0.233	<0.001 *	0.000	1.000	−0.149	<0.001 *	0.126	0.002 *
**TG**	0.344	<0.001 *	−0.052	0.192	0.126	0.002 *	−0.142	<0.001 *
**Systolic BP**	0.109	0.006 *	0.025	0.537	0.131	<0.001 *	−0.131	<0.001 *
**Diastolic BP**	0.088	0.028 *	0.012	0.764	0.131	<0.001 *	−0.101	0.012 *

* *p* < 0.05; BMI, body mass index; BP, blood pressure; HOMA-IR, Homeostatic Model of Assessment Insulin Resistance; HOMA-β, Homeostatic Model of Assessment β-cell function; HDL, high-density lipoprotein; TG, triglycerides.

**Table 9:** Associations between regional adiposity and metabolic characteristics in medicated participants.

N = 1407	Visceral		Arm		Trunk		Leg	
	r	*p*-Value	R	*p*-Value	r	*p*-Value	r	*p*-Value
**HOMA-IR**	0.218	<0.001 *	0.137	<0.001 *	0.009	0.753	−0.135	<0.001 *
**HOMA-β**	0.172	<0.001 *	0.164	<0.001 *	−0.014	0.622	−0.084	0.003 *
**BMI**	0.512	<0.001 *	0.130	<0.001 *	0.195	<0.001 *	0.057	0.043 *
**HDL**	−0.324	<0.001 *	0.027	0.346	−0.148	<0.001 *	0.155	<0.001 *
**TG**	0.272	<0.001 *	−0.009	0.758	0.095	<0.001 *	−0.104	<0.001 *
**Systolic BP**	0.192	<0.001 *	0.065	0.022 *	0.078	0.006 *	−0.158	<0.001 *
**Diastolic BP**	0.166	<0.001 *	0.022	0.435	0.112	<0.001 *	−0.111	<0.001 *

* *p* < 0.05; BMI, body mass index; BP, blood pressure; HOMA-IR, Homeostatic Model of Assessment Insulin Resistance; HOMA-β, Homeostatic Model of Assessment β-cell function; HDL, high-density lipoprotein; TG, triglycerides.

**Table 10:** Associations between regional adiposity and metabolic characteristics in non-medicated participants.

N = 1140	Visceral		Arm		Trunk		Leg	
	r	*p*-Value	R	*p*-Value	r	*p*-Value	r	*p*-Value
**HOMA-IR**	0.283	<0.001 *	0.102	<0.001 *	0.000	0.997	−0.047	0.143
**HOMA-β**	0.128	<0.001 *	0.075	0.019 *	−0.004	0.909	0.006	0.843
**BMI**	0.524	<0.001 *	0.011	0.731	0.191	<0.001 *	0.019	0.548
**HDL**	−0.291	<0.001 *	0.133	<0.001 *	−0.126	<0.001 *	0.076	0.017 *
**TG**	0.375	<0.001 *	0.032	0.317	0.016	0.623	−0.060	0.060
**Systolic BP**	0.161	<0.001 *	−0.049	0.126	0.092	0.004 *	−0.101	0.002 *
**Diastolic BP**	0.166	<0.001 *	0.020	0.534	0.066	0.038 *	−0.081	0.011 *

* *p* < 0.05; BMI, body mass index; BP, blood pressure; HOMA-IR, Homeostatic Model of Assessment Insulin Resistance; HOMA-β, Homeostatic Model of Assessment β-cell function; HDL, high-density lipoprotein; TG, triglycerides.

## Data Availability

The datasets analyzed for this study can be found in the Figshare repository (https://figshare.com/articles/CODING_DATA/9642098 access date: 5 November 2019).
